# Post-Operative Radiotherapy in Prostate Cancer: Is It Time for a Belt and Braces Approach?

**DOI:** 10.3389/fonc.2021.781040

**Published:** 2021-11-18

**Authors:** Nicolas Giraud, Nicolas Benziane-Ouaritini, Ulrike Schick, Jean-Baptiste Beauval, Ahmad Chaddad, Tamim Niazi, Mame Daro Faye, Stéphane Supiot, Paul Sargos, Igor Latorzeff

**Affiliations:** ^1^ Radiation Oncology Department, Institut Bergonié, Bordeaux, France; ^2^ Radiation Oncology Department, University Hospital, Brest, France; ^3^ Urology Department, Clinique la Croix du Sud, Toulouse, France; ^4^ School of Artificial Intelligence, Guilin University of Electronic Technology, Guilin, China; ^5^ Division of Radiation Oncology, Department of Oncology, McGill University, Montreal, QC, Canada; ^6^ Radiation Oncology Department, Institut de Cancérologie de l’Ouest, Nantes Saint-Herblain, France; ^7^ Radiation Oncology Department, Clinique Pasteur, Toulouse, France

**Keywords:** prostate cancer, radiotherapy, radical prostatectomy, biochemical progression, androgen deprivation therapy

## Abstract

Approximately 30% of patients treated with radical prostatectomy (RP) for prostate cancers experience biochemical recurrence (BCR). Post-operative radiation therapy (RT) can be either offered immediately after the surgery in case of aggressive pathological features or proposed early if BCR occurs. Until recently, little data were available regarding the optimal RT timing, protocol, volumes to treat, and the benefit of adding androgen deprivation therapies to post-operative RT. In this review, we aim to pragmatically discuss current literature data on these points. Early salvage RT appears to be the optimal post-operative approach, improving oncological outcomes especially with low prostate-specific antigen (PSA) levels, as well as sparing several unnecessary adjuvant treatments. The standard RT dose is still 64–66 Gy to the prostate bed in conventional fractionation, but hypofractionation protocols are emerging pending on late toxicity data. Several scientific societies have published contouring atlases, even though they are heterogeneous and deserve future consensus. During salvage RT, the inclusion of pelvic lymph nodes is also controversial, but preliminary data show a possible benefit for PSA > 0.34 ng/ml at the cost of increased hematological side effects. Concomitant ADT and its duration are also discussed, possibly advantageous (at least in terms of metastasis-free survival) for PSA rates over 0.6 ng/ml, taking into account life expectancy and cardiovascular comorbidities. Intensified regimens, for instance, with new-generation hormone therapies, could further improve outcomes in carefully selected patients. Finally, recent advances in molecular imaging, as well as upcoming breakthroughs in genomics and artificial intelligence tools, could soon reshuffle the cards of the current therapeutic strategy.

## Introduction

Radical prostatectomy (RP) is one of the standard treatment options for localized prostate cancers (PCa). However, after RP, the 5-year risk of biochemical recurrence (BCR) nears 30% (1). As a matter of fact, 10%–40% of patients treated with RP will be offered additional radiotherapy (RT), either adjuvant (immediately after the surgery), or in a salvage setting following a PSA increase, characterizing BCR (2).

Considering published adjuvant RT randomized trials (3–6) as well as recent studies comparing adjuvant and salvage strategies (7–10), some factors predictive of BCR have been identified, leading to a change of paradigm to better personalize post-operative management. Similarly, the indications of androgen deprivation therapy (ADT) associated with post-operative RT have been more precisely defined (11–13), and ongoing trials even explore treatment intensification (for instance, with new-generation hormone therapies) for patients harboring unfavorable prognostic factors.

In this review, we aim to discuss current literature data, present pragmatic advice on how and when to treat PCa patients with RT in the post-operative setting, and offer new perspectives as to the personalization of therapeutic strategies after RP.

## Optimal Post-Operative Treatment Timing: Is Sooner the Better?

The optimal timing to consider post-operative radiation is still debated. RT can be discussed immediately after the surgery, in an adjuvant setting, optimizing disease control at the potential cost of overtreatment and pelvic toxicity (3–5). Alternatively, it can be offered aloof from the surgery in case of PSA elevation, reducing the risk of overtreatment but with limited scientific proof, at least until recently.

Seven randomized controlled studies have evaluated the timing of RT after RP. During the 90s, four trials have compared adjuvant RT to observation: EORTC-22911, ARO 96-02, SWOG-8794, and FinnProstate (3–6). In these studies, adjuvant RT showed a significant increase in recurrence-free survival among high-risk patients based on pathology findings (positive surgical margins, >pT3, Gleason score 8–10) compared to observation. Metastasis-free survival (MFS) and overall survival (OS), however, were improved only in the SWOG-8794 trial. These studies offer long-term follow-ups, but salvage RT at the time of biological recurrence was not compulsory in the control arm, and, when offered (6), was often late, at a high PSA level (PSA > 0.5 ng/ml). As a reminder, post-RP BCR is currently defined by a PSA level > 0.2 ng/ml (with ongoing discussions to move onward to a 0.1 ng/ml threshold), undetectable after the surgery but showing ascending kinetics after 3 months (14). Overall, up to 40% of patients eligible to adjuvant RT according to the inclusion criteria of these trials did not present BCR after 10 years, thus theoretically not needing further treatments (3, 4). For some of these patients, adjuvant RT could have even been an overtreatment, associated with respectively 15%–35% and 2%–8% early and late grade 2 or higher genito-urinary (GU) and gastro-intestinal (GI) toxicity (4–6). Consequently, despite the results of these randomized trials, adjuvant therapeutic strategy has not been adopted in routine practice, with RT delayed until BCR, although supporting evidence for the latter has been lacking until recently (7–9).

Three randomized phase III trials (RADICALS-RT, RAVES and GETUG-AFU 17) have compared adjuvant and early salvage RT at BCR for patients with a significant risk of recurrence (**Table 1**) (7–9). RADICALS (Radiotherapy and Androgen Deprivation In, Combination After Local Surgery) randomized 1,396 patients into two parts: one exploring the timing of post-operative RT (RADICALS-RT) and the other addressing the optimal duration of post-operative ADT. In RADICALS-RT, patients were randomized between adjuvant RT or observation followed by salvage RT at the time of BCR (defined as a PSA ≥0.1 ng/ml or three consecutive rises). Two prostate bed RT schedules were authorized: 66 Gy in 33 fractions or 52.5 Gy in 20 fractions. With a median follow-up of 5 years, the biochemical recurrence-free survival (BRFS) was 85% in the salvage RT group, *versus* 88% in the adjuvant RT group [hazard ratio (HR) 1.10; *p* = 0.56]. Self-reported urinary incontinence was worse at 1 year for those in the adjuvant RT arm (*p* = 0.0023). Grade 3–4 urethral stricture within 2 years was reported in 6% of patients in the adjuvant RT arm *versus* 4% in the salvage RT arm (*p* = 0.02) and the data are maturing for distant MFS assessment (7).

**Table 1:** Results from prospective trials comparing adjuvant and salvage RT.

	Study design	Patient number	Eligibility criteria	Treatment arms	Primary endpoint	GU late toxicity
RADICALS-RT (7)	Superiority	1,350	Positive margin or pT3a/pT3b/pT4 or Gleason 7-10	Adjuvant *versus* early salvage RT	BRFS 85% *versus* 88% (*p* = 0.56)	Grade ≥ 3 urethral stenosis 6% *vs*. 4% (*p* = 0.02)
RAVES (8)	Non inferiority	424	Positive margin or pT3a/pT3b	Adjuvant *versus* early salvage RT	BRFS 86% *versus* 87% (*p* = 0.15)	Grade ≥ 2 70% *vs*. 56% (*p* < 0.001)
GETUG-17 (9)	Superiority	333	Positive margin and pT3a/pT3b/pT4	Adjuvant RT + ADT *versus* early salvage RT + ADT	BRFS 92% *versus* 90% (*p* = 0.42)	Grade ≥ 2 59% *vs*. 22% (*p* < 0.0001)

RT, radiotherapy; BRFS, biochemical recurrence-free survival; GU, genito-urinary; G, grade; ADT, androgen deprivation therapy.

The RAVES study is a non-inferiority phase III trial with 333 patients harboring incomplete resection margin (R1) and/or pT3 PCa randomized between 64 Gy adjuvant prostate bed-only RT (0 to 4 months post-RP) or the same protocol but only when PSA levels reached ≥0.2 ng/ml. With 50% of patients receiving salvage RT triggered by PSA levels, 5-year freedom from biochemical progression was 86% in the adjuvant RT group *versus* 87% in the salvage RT group, not meeting the protocol pre-specified criteria for non-inferiority. Nevertheless, grade 2 or higher GU toxicity was lower in the salvage RT group (54% *versus* 70%), and grade 2 or higher GI toxicity was similar (10% *versus* 14%) between arms. Quality-of-life data should be published soon. Patient-reported outcomes regarding GU and GI toxicity, assessed by standardized questionnaires, were similar, except for a small increase in 1-year urinary and fecal incontinence symptoms in the adjuvant RT arm, but resolving after a longer follow-up (8).

The GETUG-AFU 17 trial randomized 424 PCa patients with pT3-4 and/or R1 pNx (without pelvic lymph nodes dissection), or pN0 (with negative lymph nodes dissection) disease between immediate and early salvage RT, both combined with 6 months of ADT. With a median follow-up of 75 months, event-free survival (either clinical, biochemical, or death) at 5 years was 92% in the adjuvant RT arm, *versus* 90% in the salvage RT arm (HR = 0.81, *p* = 0.42). Grade 2 or higher GU toxicity rate was significantly higher in the adjuvant RT arm (27% *versus* 7%, *p* > 0.0001), as well as erectile dysfunction (*p* < 0.0001). Long-term quality-of-life assessments did not differ between the groups (9).

Finally, the ARTISTIC meta-analysis aggregated the data from these three studies, gathering 2,153 patients and 270 events. It failed to bring evidence that adjuvant RT outperformed early salvage RT in event-free survival (89% *versus* 88%) (10). The authors concluded that until data on long-term outcomes are available, early salvage RT would seem to be the preferable treatment policy as it offers the opportunity to spare many men from RT and its associated side effects.

In case of low stable post-operative PSA, benign glandular tissue at the margins might be the explanation. In this situation, a review of pathological specimen seeking benign margins, particularly at the bladder neck, could be useful. However, overall, benign prostatic elements remain an exceptional source of elevated PSA (15, 16).

Besides pathological data, the main prognostic factor for salvage RT response is the PSA level at the time of RT (17). Considering exclusively patients receiving early salvage RT (PSA <0.5 ng/ml), 5-year BRFS and MFS reached 63%–80% and 85%–90%, respectively (11, 18–20). A systematic review gathering more than 5,500 patients treated with salvage RT showed a BRFS loss of 2.6% per 0.1 ng/ml PSA increase, highlighting the importance of treating early (21). Consequently, current European Association of Urology (EAU) guidelines advocate for early salvage RT as soon as the 0.2 ng/ml PSA threshold is reached, without awaiting the 0.5 ng/ml level that had been found to independently impact BRFS, MFS, cancer-specific, and all-cause mortality rates (2, 22). Salvage RT at even lower PSA thresholds could be more optimal as suggested by retrospective studies (20). Of note, in the RADICALS-RT trial, PSA of 0.1 ng/ml was the chosen threshold to initiate salvage RT (7).

## Radiation Doses and Target Volumes: Should We Hit Harder and Wider?

### Doses

Historically, before the advent of intensity modulated RT (IMRT), doses of 60 Gy to the prostate bed with conventional fractionation were used in the post-operative setting (17, 23). Currently, slightly higher doses of 64–66 Gy are prescribed and were used in the recently published salvage trials (10).

Guided by advances in RT allowing for more precise and less toxic treatments, such as image-guided RT (IGRT), dose escalation has been explored and has been shown to be potentially associated with BRFS (24). The recently published European SAKK 09/10 is a multicentric randomized phase III trial for PCa patients presenting with BCR (defined as two consecutive rises with a last PSA value > 0.1 ng/ml or three consecutive rises) after RP with a PSA nadir of ≤ 0.4 ng/ml and a PSA ≤ 2 ng/ml at randomization. Its goal was to evaluate the benefit of dose-intensified RT (70 Gy in 35 fractions) compared to conventional-dose RT (64 Gy in 32 fractions) given to the prostate bed. The absence of macroscopic disease or nodal involvement at RP was required, and both IMRT or 3D-RT were allowed. After a median follow-up of 6.2 years, with 350 patients randomized and a median pre-RT PSA value of 0.3 ng/ml (range, 0.03–1.61 ng/ml), the estimated 6-year freedom from biochemical failure was 62.3% [95% confidence interval (CI) 54.2%–69.4%] in the 64 Gy arm, *versus* 61.3% (95% CI 53.4%–68.3%) in the 70 Gy arm, without any significant difference between arms (hazard ratio [HR] 1.14, 95% CI 0.82–1.60, *p* = 0.44). No subgroup was identified that could benefit from this dose intensification. There was also no difference found in terms of clinical progression-free survival (PFS), time to hormone treatment, and OS. Despite early signals disfavoring dose escalation to 70 Gy in terms of GU toxicity (mean difference in score change between arms, 3.6; *p* = 0.02), final rates of grade 2 or higher GU toxicity did not significantly differ between the two groups. There was indeed no impact of dose intensification on early urinary recovery or prevalence of *de novo* incontinence. Grade 2 and 3 GI symptoms were significantly more common with dose-escalated RT (20% and 2.3%, respectively, *versus* 7.3% and 4.2%; *p* = 0.009), but quality-of-life indicators were not altered with dose intensification (25).

In the post-operative setting, another study aiming to assess dose escalation was published in 2020 by Qi et al. This study randomized 144 patients with pT3-4, positive surgical margins, or rising PSA ≥0.2 ng/ml after RP to either 66 Gy in 33 fractions or 72 Gy in 36 fractions; prophylactic pelvic irradiation was also delivered in high-risk patients. The primary endpoint, BRFS, was similar between groups after a median follow-up of 48.5 months (75.9 *versus* 82.6%, *p* = 0.299). While patients with higher Gleason scores (8-10) may have benefited most from the intensification (BRFS of 79.7% *versus* 55.7%; *p* = 0.049), randomization was not stratified and ADT was not allowed in the study. Finally, no difference was found in terms of acute or late grade 2 or higher GI or GU toxicities (26).

Based on these results, dose escalation beyond 66 Gy does not seem to offer any improvement of oncological outcomes, at the cost of increased grade 2 or higher toxicity. Soon, the advent of novel radiotracers may guide further refinement of treatment volumes and dose escalation focused exclusively on the residual disease targets (27, 28).

### Fractionation

Hypofractionation protocols, taking advantage of the low α/β ratio of PCa, are validated for the initial management of localized PCa (29). In the post-operative setting, a few retrospective series evaluating moderate hypofractionation have shown good biochemical control rates with low rates of acute toxicity, but concerns regarding late side effects remain (30). Indeed, post-operative tissues could hold impaired repair abilities due to radiation-induced urothelial damages. Thus, in 247 patients treated with RT using several hypofractionated regimens, Cozzarini et al. reported 18.1% grade 3 GU late toxicities. Some patients received a high dose, 71.4 Gy in 28 fractions (equivalent dose in 2 Gy fractions of 79.2 Gy assuming an α/β ratio of 3) and others received pelvic RT potentially explaining this higher than expected rate of grade 3 or higher late toxicities (31). In contrast, other studies are more reassuring, for instance, Franceze et al. reporting grade 3 late GU and GI toxicity rates of 2.7% and 1.6% at 5.5 years using 70 Gy (65–74.2) in 25–28 fractions (32). These prospective data regarding the safety of such regimen have been emerging and most of them show reassuring safety data. For example, in a phase II study of 61 patients treated with 51 Gy in 15 fractions using IMRT and IGRT, with a limited median follow-up of 16 months, 11.5% and 13.1% of patients experienced acute grade 2 or higher GU and GI symptoms, respectively. The late grade 2 or higher GU toxicity rate was 8.2%, and late grade 2 or higher GI toxicity rate was 11.5% without any grade 3 GI adverse event being reported (33). On the other hand, Lewis et al. reported 28% grade 3 or higher late GU toxicity and 7% persisting hematuria in 56 patients treated with 65 Gy in 26 fractions, possibly due to the large volume and the high proportion of patients on anticoagulants (34). Regarding oncologic outcomes, Chin et al. reported long-term disease control similar to conventional fractionation at 10 years for 112 men treated with moderate hypofractionation (55.5 Gy in 20 fractions, EQD2 61.9 Gy with α/β = 3) (35). In summary, moderately hypofractionated RT (<3 Gy per fraction) delivered with novel RT techniques (IMRT, image-guided RT) is still not ready for prime time, pending on more mature reports of late toxicity. Indeed, with contrasting late toxicity reports so far, prospective trials are still warranted before wide adoption. NRG GU003 (NCT03274687) is one of these upcoming trials, comparing 66.5 Gy in 25 fractions *versus* 66.6 Gy in 37 fractions. The previously cited RADICALS-RT trial could also contribute to more data on this topic, as both 66 Gy in 33 fractions and 52.5 Gy in 20 fractions were allowed (7).

For extreme fractionation, only very preliminary data exist; thus, it should not be used outside of clinical trials. Difficulties arise notably in the prostate bed deformation and the proximity of organs at risk. In this setting, Yoon et al. recently reported the outcome of 18 patients treated with 5 fractions of 6–6.8 Gy to the prostatic bed. Close image monitoring prior to and during each T session as well as strict bladder and rectum filling preparations highlighted the importance of considering clinical target volumes (CTV) deformation in order to avoid CTV misdosing (36). Regarding the tolerance of extreme fractionation, a phase I escalation trial by Sampath et al. tested three dose levels (35 Gy, 40 Gy, and 45 Gy in 5 fractions, given every other day) in 23 patients. With a follow-up of 60 months, no acute dose-limiting toxicity was observed. Grade 3 late GU toxicity was reported in four patients (two urethral stenosis and two incontinence) between 30 and 38 months, two patients having been treated by the 45-Gy protocol (37).

When treated, pelvic lymph nodes should receive a dose range of 45–50.4 Gy with conventional fractionation similarly to primary prostate RT, according to the latest NRG recommendations (38). In this setting, we did not find any prospective evidence of hypofractionation protocols for pelvic RT; thus, it should not be administered outside clinical trials. Overall, the standard post-operative RT protocol remains conventional fractionation, with moderate hypofractionation regimens progressively gaining interest pending on late toxicity data. Stereotactic RT is still highly investigational and should, for now, be limited to clinical trials.

### Volumes

Post-operative CTV typically includes the prostate bed, with or without pelvic lymph nodes. The definition of the prostate bed is, however, subject to important intra- and inter-observer variability. Numerous recommendations have been published to reduce these fluctuations, but several differences emerge between them, notably in the definition of the apex or base limits, making it difficult to reach an international consensus (39–42). CTV delineation can furthermore be impacted by the newest imaging techniques such as Choline or PSMA PET/computed tomography (CT) or MRI (43). Recently, the Groupe Français de Radiothérapie Urologique (GFRU) has offered updated recommendations associated with a contouring atlas based on anatomical boundaries reproducible on planning CT (44). The GFRU contouring guidelines were validated in a secondary analysis from a prospective trial. Indeed, these guidelines were evaluated in 30 patients presenting patterns of local recurrence on 18F-fluciclovine PET/CT. Overall, 90% of recurrences were covered, and 10% were missed or marginally covered (45).

In 20%–50% of relapsing patients, a local recurrence is responsible for the BCR (46, 47). Surgical data and the advent of new radiotracers have shown that pelvic nodal recurrences are more common than previously believed, highlighting the need to better explore pelvic lymph-node irradiation (48). The RTOG 0534 SPPORT trial is a randomized three-arm phase III study designed to estimate the input of adding short-term ADT to post-operative prostate bed RT (PBRT) on 5-year freedom from progression (defined as a PSA crossing nadir + 2 ng/ml, clinical failure or death from any cause), and on the other side whether the addition of pelvic node RT (PNRT) to short-term ADT and prostate bed RT is beneficial. Patients were included if they had pT2-T3 and/or pN0-x and/or Gleason score <9 M0 disease, and a PSA between 0.1 and 2.0 ng/ml at least 45 days post-RP. Radiation doses were 64.8–70.2 Gy given to the prostate bed and 45 Gy to a pelvic lymph node CTV, all in 1.8-Gy fractions. In the interim analysis of the 1,191 patients included, after a median follow-up of 5.4 years, freedom from progression rates was 71.1% for PBRT alone, 82.7% for PBRT+ADT, and 89.1% for PBRT+ADT+PNRT, demonstrating a 6.4% benefit with the use of PNRT (*p* = 0.0063, HR 0.71; 95% CI 0.51–0.98). There was no significant difference regarding the incidence of distant metastases (HR 0.64, 95% CI 0.39–1.06), to be confirmed through a longer follow-up. Side effects were similar among the different groups, with 4.9% and 6.0% late grade 3 or higher GU toxicity, and 0.4% and 1.1% late grade 3 or higher GI toxicity in the PBRT + ADT + PNRT *versus* PBRT + ADT arms, respectively. The main additional severe side effect from PNRT was hematological, with more grade 2 or higher (5.1%, versus 2.3% with PBRT alone) and grade 3 or higher (2.6%, *versus* 0.5% with PBRT alone) blood-bone marrow adverse events. From this study, PNRT and ADT additions to PBRT seem to offer biochemical control benefits especially for patients with PSA ≥ 0.34 ng/ml. The updated results with prolonged follow-up will help further uncover the magnitude of the benefit of pelvic radiation in this setting (13).

## Should Concomitant Androgen-Deprivation Therapy Be Compulsory?

Androgen receptors are known stimulators of DNA repair genes (49). Hormone therapy acts as a radiosensitizer, disabling androgen receptor-mediated reparation of DNA damages caused by RT in PCa cells, thus decreasing the ability of cancer cells to recover from radiation damages (50). In the adjuvant and salvage settings, the rationale behind ADT use could be to fight subclinical micro-metastases. High-level evidence indicates for ADT use for unfavorable intermediate- and high-risk PCa combined with RT when used as primary treatment, improving overall survival in these cases (2). However, historically, ADT benefits in the setting of post-operative RT have been controversial.

In the adjuvant setting, there is to date no prospective trial supporting exclusive or concomitant ADT use. Answers could be given by ongoing randomized prospective trials (51). Among these, the EORTC 22043-30041 trial (NCT00949962) aimed to evaluate the addition of 6 months of leuprorelin to adjuvant RT for patients stratified according to the T-stage (pT2R1 versus pT3R0 *versus* pT3R1) and the Gleason score (≤ 3 + 4 *versus* ≥ 4 + 3).

In the salvage RT setting, two randomized controlled studies have explored the addition of ADT. The GETUG-AFU-16 trial randomized 743 PCa patients with biochemical recurrence in two groups: RT alone *versus* RT plus 6 months of Goserelin. All patients had undetectable post-operative PSA levels (<0.1 ng/ml) following RP. The 10-year (clinical and biochemical) PFS was 64% (95% CI, 58–69) for patients treated with Goserelin and RT, and 49% (95% CI, 43–54) with RT alone (HR 0.54, 95% CI 0.43–0.68, log-rank test *p* < 0.0001). The respective 10-year MFS were 75% (95% CI, 70–80) and 69% (95% CI, 63–74), which was statistically significant (HR 0.73, 95% CI 0.54–0.98, log-rank test *p* = 0.034). There was no difference regarding late toxicity between arms (11). These results are supported by the RTOG-9601 trial. In this phase III double-blind trial, 760 patients with BCR or with post-operative detectable PSA (>0.1 ng/ml) were randomized between salvage RT alone or with 2 years of bicalutamide at 150 mg daily. After a median follow-up of 12 years, OS was 5% higher in the salvage RT plus bicalutamide group (HR 0.77, *p* = 0.04) with an even stronger effect when pre-therapeutic PSA was >1.5 ng/ml (HR 0.45, *p* < 0.007). Prostate cancer-specific mortality and MFS were significantly lower with the combined treatment (HR 0.49 and 0.63 respectively, *p* < 0.01). Grade 3–4 late toxicity was similar between groups. However, 70% of patients treated with bicalutamide reported gynecomastia, versus 11% in the RT alone group (*p* < 0.001) (12). With these results, anti-androgens alone are no longer approved by the Food and Drug Administration (FDA) in the United States (52). Thus, gonadotropin-releasing hormone (GnRH) agonists or antagonists should be favored.

The optimal ADT duration is still debated when associated with salvage RT. Extrapolating from the RTOG-9601 and GETUG-16 results remains uneasy; none of these trials being exclusively on the early intervention of post-operative RT defined as PSA < 0.5 ng/ml (**Table 2**).

**Table 2:** Results from prospective randomized trials comparing salvage RT with or without ADT.

	Patient number	Eligibility criteria	Treatment arms	MPFS	BRFS
GETUG-16 (11)	743	pT2-pT4 N0 Rising PSA >0.2–2 ng/ml	RT *versus* RT + A-LHRH 6 months	At 10 years : 69% *versus* 75% (*p* = 0.034)	At 5 years : 62% *versus* 80% (*p* < 0.0001)
RTOG-9601 (12)	760	pT2R1N0, pT3N0, Detectable PSA (0.2–4 ng/ml)	RT *vs*. RT + ADT 2 years	At 12 years: 77% *versus* 85.5% (*p* = 0.005)	At 12 years : 32.9% *versus* 56% (*p* < 0.001)

RT, radiotherapy; A-LHRH, luteinizing hormone-releasing hormone antagonists; MPFS, metastasis progression-free survival; BRFS, biochemical recurrence-free survival; ADT, androgen deprivation therapy.

Moreover, updated data from RTOG-9601 have shown that the pre-salvage RT PSA level could be a prognostic biomarker, guiding the use of ADT with RT. In patients receiving late salvage RT (PSA > 1.5 ng/ml), 2 years of ADT were associated with increased OS (HR 0.45, 95% CI 0.25–0.81, *p* = 0.01). For PSA levels between 0.6 and 1.5 ng/ml, 2 years of ADT were associated with a better MFS only (HR 0.67, 95% CI 0.47–0.95, *p* = 0.03). Finally, for patients receiving early salvage RT (PSA ≤ 0.6 ng/ml), no difference was found regarding OS when adding 2 years of ADT (HR 1.16, 95% CI 0.79–1.70). Furthermore, these patients presented increased mortality from all causes [odds ratio (OR) 1.94, 95% CI 1.17–3.20, *p* = 0.01] and higher rates of grade 3–5 cardiac and neurological toxicities (OR 3.57, 95% CI 1.09–15.97, *p* = 0.05), highlighting the importance of considering the competitive risk between PCa and cardiovascular comorbidities when using these treatments (53). ADT-related toxicity is suspected to be linked with its duration (17). The RADICALS-HD trial (NCT00541047) is currently randomizing patients between 6 months, 24 months, and no GnRH agonist. It will help clarify the optimal ADT duration in the post-operative setting. The LOBSTER trial (NCT04242017), a Belgian phase II randomized multicentric study, is also comparing 6 months *versus* 24 months of ADT in case of salvage RT for BCR after pN0 PCa.

## Perspectives: What’s Up, Doc?

### New-Generation Imaging-Guided RT

At the time of post-operative BCR determined by the current definitions, PSA levels are still too low to offer good sensitivity on standard imaging (computed tomography or bone scintigraphy) (54). Dynamic Magnetic Resonance Imaging (MRI) allows the detection of ≥5-mm lesions for PSA levels <2 ng/ml, with a negative predictive value of 95% (55). PET/CT with new Choline and PSMA radiotracers could be increasingly used to assess post-operative recurrence. Choline-PET, in prospective trials, holds 50%–78% detection rates for PSA levels between 0.5 and 1 ng/ml (56). In the recently published phase II/III Emory Molecular Prostate Imaging for Radiotherapy Enhancement (EMPIRE-1) monocentric trial, 3-year event-free survival was significantly improved by 12% (*p* = 0.0028) when using PET-Choline to guide the delineation of RT volumes, with similar toxicity (25). Regarding PET-PSMA, prospective studies have confirmed its overall good detection ability when PSA levels reached the ideal therapeutic range for salvage RT, i.e., 0.2–0.5 ng/ml, with 22%–58% detection, reaching 57%–71% for PSA between 0.5 and 1 ng/ml (increasing along with PSA levels) (56).

In prospective trials, PSMA-PET motivated a change of patient care more than half of the times (57). However, it remains unanswered whether these changes based on PSMA “re-staging” impact oncological outcomes. In the context of post-operative RT, the PSMA-SRT (NCT03582774) phase III trial is the first to evaluate the impact of the molecular imaging for patients presenting with post-operative BCR. This study is randomizing patients between conventional and PET-PSMA imaging before salvage RT planning with BRFS as primary endpoint. The first results are expected in 2024 (58).

### Treatment Intensification

In prostate cancer, an increasing number of trials aim to bring lines of treatments further upstream, hoping to improve long-term outcomes. Thus, intensification strategies are on the lookout, for example, with the addition of 6 months of apalutamide, a new-generation hormone therapy, to ADT and salvage RT in the randomized phase III CARLHA 2 GETUG 33 trial (NCT04181203) for patients showcasing high-risk BCR (at least one of the following characteristics: PSA > 0.5 ng/ml, Gleason > 7, pT3b, PSA doubling time < 6 months). Apalutamide is also tested in the NRG GU006 study (NCT03371719), a two-arm phase II trial testing in this setting RT alone or with apalutamide. In a similar approach, ANZUP1801 is exploring the addition of 96 weeks of darolutamide to RT and ADT, either for primary definitive therapy or in an adjuvant setting for very high-risk PCa (NCT04136353), and STEEL is studying the addition of 2 years of Enzalutamide with salvage RT and 2 years of ADT when aggressive features are displayed (NCT03809000).

### Personalized Medicine

Patient selection is crucial to identify which patients could benefit most from intensification or de-intensification treatment strategies. In the future, apart from usual factors with decisional influence (pathological data, comorbidities, patient choice, etc.), new types of data are emerging, which could impact the therapeutic strategy.

In that way, personalized approaches are being evaluated using genomics. For instance, the DECIPHER test, a tissular test using a 22-gene molecular signature, could predict BCR and cancer-specific mortality, and impact adjuvant treatment decisions (59, 60). In an ancillary study of the RTOG-9601 trial, this score was generated from 352 of 760 randomized patients with a median follow-up of 13 years. On multivariable analysis, the genomic classifier (GC) was independently associated with the risk of distant metastasis (HR 1.17, 95% CI 1.05–1.32; *p* = 0.006), PCa-specific mortality (HR 1.39, 95% CI 1.20–1.63; *p* < 0.001), and OS (HR 1.17, 95% CI 1.06–1.29, *p* = 0.002) after adjusting for age, ethnicity, Gleason score, T stage, margin status, initial PSA, and treatment arm (61). The 4Kscore blood test can also be mentioned, awaiting its Clinical Laboratory Improvement Amendment (CLIA) accreditation. It aims to predict the tumoral aggressiveness on the prostatectomy specimen, thus guiding adjuvant radiotherapy indications (62).

Simultaneously, artificial intelligence-guided tools are rising, taking into account large volumes of heterogeneous data to produce effective prediction models. For instance, using multicentric data from 5,043 patients, a deep learning model was able to predict 3-year BCR with an area under the curve of 0.70 (0.84 when adding post-operative data), surpassing the more conventional CAPRA-S recurrence risk score (AUC 0.63) (63). This approach could allow closer monitoring and early treatment of high-risk patients as pre-defined by the model.

A selection of ongoing recruiting trials exploring these emerging fields applied to post-operative PCa can be found in **Table 3**.

**Table 3:** Selection of ongoing trials regarding treatment intensification and image-guided RT for post-operative prostate cancer.

NCT number	Phase	Country	Estimated enrollment	Situation	Arms	Primary outcome
NCT04242017	2	Belgium	394	Salvage post-RP	70 Gy/35 fr sRT + 6-month ADT 70 Gy/35 fr sRT + 24-month ADT	MFS
NCT04642027	3	Netherlands	538	Salvage post-RP	70 Gy/35 fr PSMA-guided sRT 60 Gy/20 fr PSMA-guided sRT	5-year PFS (biochemical, clinical, loco-regional or distant progression, ADT use)
NCT04794777	3	Sweden	450	Salvage post-RP	Standard sRT PSMA-guided sRT	Biochemical PFS
NCT03582774	3	USA	193	Salvage post-RP	Standard sRT PSMA-guided sRT	Biochemical PFS
NCT04136353	3	Australia	1,100	Definitive or salvage RT	Standard RT + ADT Standard RT + ADT + 96 weeks of Darolutamide	MFS
NCT04181203	3	France	490	Salvage post-RP	Standard sRT + 6-month ADT Standard sRT + 6-month ADT + 6-month Apalutamide	PFS (loco-regional or distant recurrence, or death)
NCT03371719	2	USA	311	Salvage post-RP	Standard sRT + 6-month placebo Standard sRT + 6-month Apalutamide	PFS (biochemical, clinical or radiographic, or death)
NCT03899077	2	Belgium	202	Salvage post-RP	Standard sRT + 24-week ADT Standard sRT + 6-month Apalutamide	Sexual score
NCT03809000	2	USA	242	Salvage post-RP	66-70.2 Gy standard sRT + 24-month ADT 66-70.2 Gy standard sRT + 24-month ADT + 24-month Enzalutamide	PFS (progression or death)

## Conclusion

There is still a gray zone regarding the optimal salvage treatments for PCa patients after prostatectomy. Early salvage RT appears to be the best option for patients showing post-operative BCR, when administered as early as possible after crossing the current recurrence threshold of 0.2 ng/ml, which could soon be lowered to 0.1 ng/ml in light of recent data. For patients at a higher risk of relapse, especially PSA-driven, concomitant ADT seems beneficial (with ADT use associated with improved MFS outcomes when exceeding 0.6 ng/ml rates), even though results from ongoing prospective trials are still awaited to determine its optimal duration, as well as the possible benefit of treatment intensification with new hormonal agents. Recent advances in metabolic imaging, notably with the democratization of PSMA-PET, are bound to refine future treatment strategies, pending on its impact on oncological outcomes. Finally, breakthrough of genomics and artificial intelligence tools could play a key role in the upcoming years in defining the best therapeutic strategies for our patients, with an aim of personalized medicine.

## Data Availability Statement

The original contributions presented in the study are included in the article/supplementary material. Further inquiries can be directed to the corresponding author.

## Author Contributions

Conceptualization: NG, PS, and IL. Data curation: NB-Q and NG. Formal analysis: All authors. Funding acquisition: Not applicable. Investigation: NG, NB-Q, and PS. Methodology: NG, NB-Q, and PS. Project administration: IL and PS. Resources: IL and PS. Software: IL and PS. Supervision: PS. Validation: All authors. Visualization: All authors. Writing—original draft: NG and NB-Q. Writing—review and editing: All authors. All authors contributed to the article and approved the submitted version.

## Conflict of Interest

The authors declare that the research was conducted in the absence of any commercial or financial relationships that could be construed as a potential conflict of interest.

## Publisher’s Note

All claims expressed in this article are solely those of the authors and do not necessarily represent those of their affiliated organizations, or those of the publisher, the editors and the reviewers. Any product that may be evaluated in this article, or claim that may be made by its manufacturer, is not guaranteed or endorsed by the publisher.
